# Role of interfacial mode coupling of optical phonons on thermal boundary conductance

**DOI:** 10.1038/s41598-017-10482-z

**Published:** 2017-09-08

**Authors:** Ashutosh Giri, Patrick E. Hopkins

**Affiliations:** Department of Mechanical and Aerospace Engineering, University of Virginia, Charlottesville, Virginia, 22904 USA

## Abstract

We investigate the influence of optical phonon coupling across interfaces comprised of different materials with varying crystallographic orientations on the overall thermal boundary conductance. We show that for interfaces formed between a fcc solid and a L1_0_ solid (where L1_0_ solids exhibit alternating atomic layers in certain orientations), coupling between acoustic phonons in the fcc crystal and optical phonons on the L1_0_-side of the interface leads to a highly anisotropic thermal boundary conductance, where optical phonons can considerably enhance the conductance in a preferred crystallographic orientation of the layered solid. We attribute this in part to directionally dependent group velocities of optical phonons in the different crystallographic directions. For interfaces comprised of materials exhibiting diamond cubic crystal structures, higher conductances are observed for interfaces where there is a better overlap of acoustic phonons on either side of the interface, whereas, acoustic phonons directly coupling with high frequency optical phonons is shown to lower the overall conductance, especially at high temperatures where anharmonic interactions become important. Unique to the interfaces formed between the materials with diamond cubic crystal structures studied in this work, the presence of localized interfacial optical modes mediate thermal conductance across these interfaces.

## Introduction

In most modern-day nanoscale devices, thermal transport is limited by the high densities of interfaces rather than the materials that comprise the device^1, 2^. The relation between the nanoscale temperature discontinuity, Δ*T*, at the interface between the materials and the impinging heat flux, *Q*, at the interface quantifies the thermal boundary conductance (*h*_K_ = *Q*/Δ*T*)^3^, which is a measure of the efficacy with which heat propagates across the interface. Over the past few decades, *h*_K_ has been shown to be mostly limited by the acoustic impedance and mismatch of phonon spectra between the materials comprising the interface, to a first approximation^4–11^. Moreover, prior works have also demonstrated the influence of extrinsic factors such as defect concentration and roughness around the interface^2, 6, 12–16^, strength of cross-species interaction and chemistry around the interface^17–22^ and the relative crystallographic orientations of the two materials comprising the interface^23–25^ in dictating interfacial heat flow. However, despite these advances in understanding interfacial heat flow, relatively few studies have focused on understanding the role of optical phonons on thermal boundary conductance at solid-solid interfaces, even though optical phonons can make up to 90% of the available vibrational modes in some materials.

Historically, theoretical calculations have suggested that optical phonons do not contribute to dominant mean free paths and resulting thermal conductivity of crystalline solids due to their relatively small group velocities and short lifetimes relative to acoustic phonons^26, 27^. The advent of rigorous computational approaches such as first principles calculations, molecular dynamics simulations and solutions to the phonon Boltzmann transport equation, however, have highlighted the important contributions of optical modes on the thermal conductivity of bulk materials and nanostructures^28–34^. In this regard, surely, the understanding of the role of optical phonons on thermal boundary conductance across solid-solid interfaces will be crucial in designing and optimizing thermal transport across nanoscale devices with high densities of interfaces that are composed of semiconducting materials with optical branches making up a significant proportion of the dispersion relations. As such, theoretical models based on the diffuse mismatch theory have been reformulated to account for optical phonons, which can contribute significantly to the total thermal boundary conductance across interfaces of several representative material systems due to elastic and inelastic scattering^35, 36^.

In this work, we use molecular dynamics simulations and lattice dynamics calculations to investigate the role of optical phonons on thermal boundary conductance across interfaces comprised of materials with different crystallographic configurations. In particular, we study interfaces formed: 1) between a homogeneous fcc crystal and a layered L1_0_ crystal described by 6–12 Lennard-Jones (LJ) potentials; and 2) between a homogeneous diamond cubic crystal and a layered zincblende crystal described by the Stillinger-Weber (SW) potentials. We show that for interfaces formed between a fcc solid and a layered L1_0_ solid, optical phonons from the L1_0_ crystal preferentially couple to acoustic phonons of the monatomic crystal when the interface is comprised of the layered crystal oriented in the direction which accommodates for the higher group velocities of the optical modes (which is the in-plane direction for the layered crystal). A better overlap between these optical modes (in the in-plane direction) and the acoustic modes in the homogeneous crystal is shown to dramatically enhance the thermal boundary conductance. In contrast, interfaces comprised between diamond cubic and zincblende crystal structures demonstrate higher conductances for interfaces formed with a better overlap of acoustic phonons on either side of the interface. Moreover, at higher temperatures where anharmonic interactions become important, acoustic phonons directly coupling with high frequency optical phonons is shown to lower the overall conductance for these structures. Furthermore, the presence of localized optical modes at the interface mediate thermal conductance across these interfaces, which are unique to the diamond cubic crystal structures studied in this work.

## Methods and computational details

**Figure 1:**
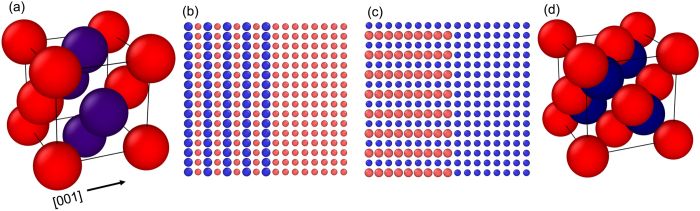
(**a**) Conventional unit cell of a diatomic L1_0_ crystal structure with a_0_ = 5.31 Å formed from materials A and B, which we refer to as an AB alloy. The atomic arrangement leads to monolayers of atoms stacked in the [001] (cross-plane) direction. Schematic of the interface formed between an AB alloy in the (**b**) cross-plane and (**c**) in-plane direction with a cubic material. (**d**) Conventional unit cell of a zincblende crystal structure (note, zincblende has two different types of atoms in its basis) with a lattice constant, a_0_ = 5.44 Å.

In a crystalline solid, a multiatom basis facilitates the presence of optical branches in the available vibrational modes; there are only three acoustic branches, while 3 *p*-3 branches of the optical variety populate the vibrational spectrum, where *p* denotes the number of atoms forming the basis^37^. In order to accommodate for optical phonons in the dispersion relation, we create a L1_0_ crystal with a four-atom basis (with two atom types occupying the basis sites that form the unit cell with a lattice parameter, *a*_0_ as shown in the schematic in Fig. 1a. The two atom types, which we refer to as material A and material B, are differentiated by mass only. The computational domains with a two point basis forms an ordered crystal that is arranged in a 1 × 1 superlattice-type structure in the[001] direction, which we refer to as the cross-plane direction (cp), whereas the[010] direction is referred to as the in-plane direction (ip); we refer to the L1_0_ crystal formed by the A and B atoms as an AB alloy. If the four basis atoms forming the unit cell in the L1_0_ structure are identical, a homogeneous fcc crystal is formed.

For the cubic fcc and L1_0_ crystals, the interactomic potential is defined by the widely used LJ potential, $U(r)=4\varepsilon [{(\sigma /r)}^{12}-{(\sigma /r)}^{6}]$, where *U* is the interatomic potential, *r* is the interatomic separation, and *σ* and *ε* are the LJ length and energy parameters, respectively. For computational efficiency, the cutoff distance is set to 2.5 *σ* for all the simulations. The length and energy parameters are modeled for LJ argon (*σ* = 3.405 Å and *ε* = 0.0103 eV, repectively) with the lattice constant *a*_0_ = 1.56 *σ*. The sizes of the computational domains are 10 *a*_0_ × 10 *a*_0_ × 80 *a*_0_ with periodic boundary conditions applied in the *x*- and *y*-directions, whereas, fixed boundaries with 4 monolayers of atoms at each end are placed in the *z*-direction. Figure 1b and c show the schematics of interfaces formed between the AB alloy and materials A and B in the in-plane and cross-plane directions, respectively.

Similarly, for the diamond cubic crystal structures, a layered domain (accommodating optical phonons and a band gap between the optical and acoustic phonons) is constructed by replacing the four basis atoms that form the unit cell with atoms of different mass (which forms a zincblende structure as shown in Fig. 1d). For the zincblende and diamond structures, all interatomic interactions are defined by the Stillinger Weber (SW) potential^38^ for Si and only the mass of the atoms are altered for simplicity. We note that as the main purpose of this work is to understand the general effect of optical modes on the thermal boundary conductance as opposed to predicting material specific properties, the use of the LJ potential for the fcc and L1_0_ crystals and SW potential for the diamond cubic and zincblende crystals will provide sufficient qualitative insights into the role of optical phonons on thermal boundary conductance across these structures.

**Figure 2:**
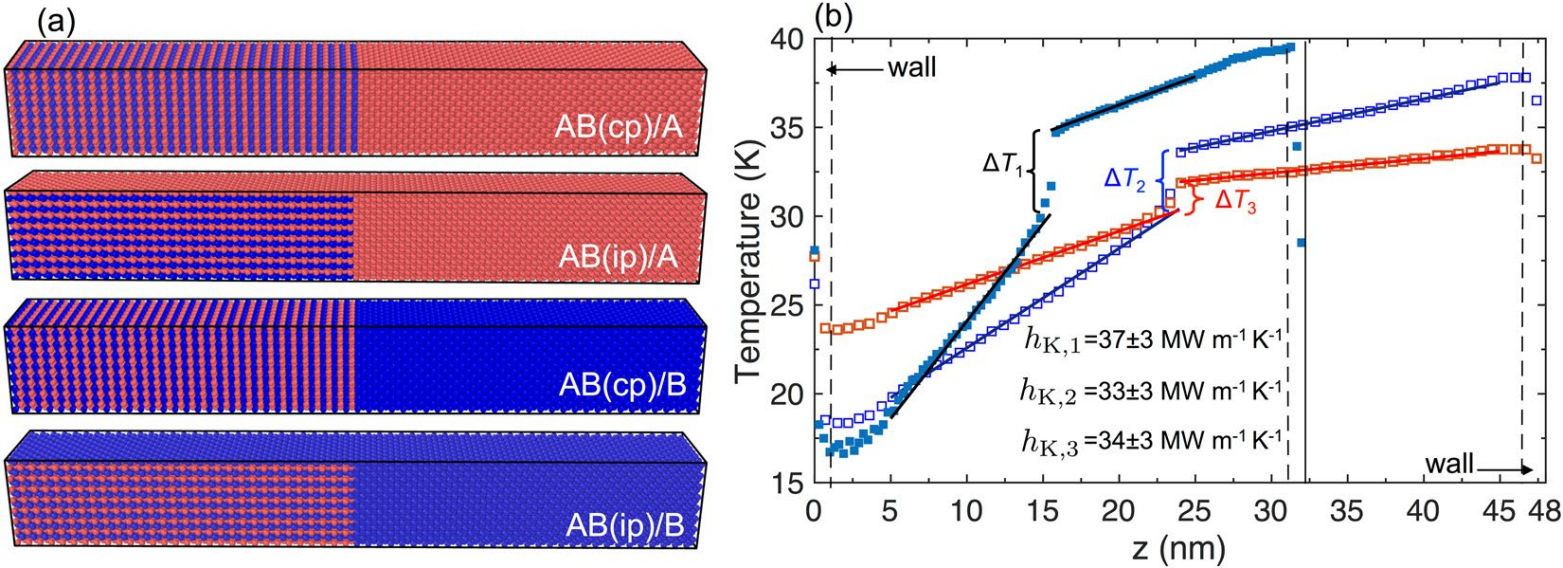
(**a**) Schematics of the computational domains formed by adjoining AB alloys oriented in the in-plane and cross plane directions with materials A and B. (**b**) Steady state temperature profiles for the AB(cp)/A computational domain with two different domain sizes along with the temperature profile calculated with the heat flux halved in comparison to the other two cases shown. The thermal boundary conductances are similar within uncertainties, suggesting that the computational domain size and the applied heat flux have negligible influence on the MD-predicted conductances.

To begin, we prescribe the masses of materials A and B to 40 g mol^−1^ (*m*_A_ = *m*_Ar_) and 160 g mol^−1^ (*m*_B_ = 4 *m*_Ar_), respectively, for the LJ-based crystals. For the thermal boundary conductance (*h*_K_) calculations, the computational domains are separated midway to create atomically smooth and perfectly matched interfaces with the difference in mass between the materials creating the acoustic mismatch. The computational domains for the AB alloy oriented in the cross-plane or in-plane directions in contact with the cubic A or B materials are shown in Fig. 2a. After the domains are setup, an equilibration scheme is imposed to generate the relaxed structures. Starting with the Nose-Hoover thermostat^39^, the number of atoms, volume and temperature of the simulation is held constant followed by the NPT integration (which is the isothermal-isobaric ensemble with the number of particles, pressure and temperature of the system held constant) for another 1 ns at 0 bar pressure. The time step for all simulations is set to 1 fs throughout the simulations.

After equilibration, a fixed amount of energy is added per time step to a warm bath at one end and removing the equal amount of energy from a cool bath at the other end (with the lengths of the baths at 10 *a*_0_ in the *z*-direction) under a microcanonical ensemble with the number of particles, volume and energy held constant. This procedure establishes a steady-state temperature gradient and the temperature of atoms in each atomic monolayer in the direction of the applied heat flux are averaged for a total of 20 ns. The temperature profiles for the first 5 ns are discarded in order for the systems to reach steady state after which the time-averaged temperature profiles are used to calculate the thermal boundary conductances. As NEMD simulations are known to exhibit size effects due to scattering of phonons with the heat baths^40, 41^, we have simulated all systems with two different sizes along the direction of the applied heat flux to make sure that our MD-predicted thermal boundary conductances are not artifacts of the simulated system sizes. Figure 2b shows the temperature profiles obtained for AB(cp)/A structures considering two different system sizes (with energy added and removed at a rate of 12.5 meV ps^−1^ from the hot and cold baths, respectively). Along with these temperature profiles, we also include the temperature profile obtained by applying a lower applied heat flux (with addition and removal of energy at a rate of 6.25 meV ps^−1^ from the heat baths). As is clear from the figure, neither the computational domain size nor the applied heat flux alter *h*_K_, which suggests that the results are independent of the system size as well the applied heat flux for our simulations. Note, to reduce the uncertainty in determining the temperature drop at the interface, linear fits to the temperature gradients are performed for the two materials on either side of the interface (excluding the nonlinear regimes near the boundaries and that comprising the heat baths).

**Figure 3:**
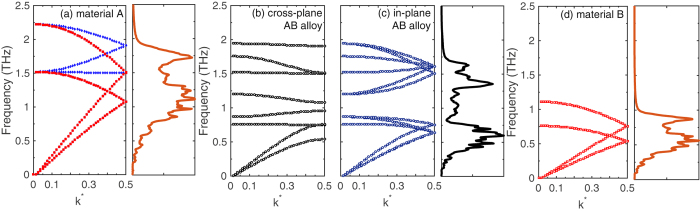
Phonon dispersion relations of (**a**) material A (*m*_A_ = 40 g mol^−1^) using both the primitive and the conventional unit cell definition to define the Brillouin zone, (**b**) AB alloy in the cross-plane and (**c**) in-plane directions and (**d**) material B (*m*_B_ = 160 g mol^−1^). Bulk phonon density of states for the structures are plotted along side the phonon dispersion relations.

The phonon dispersion relations of material A, B and the AB alloy (chosen in the direction of high symmetry) are determined via harmonic lattice dynamics calculations carried out with the General Utility Lattice Program (GULP)^42^. For the isotropic materials A and B, the Brillouin zone (represented by the truncated octahedron) is analyzed in the[001] direction, which corresponds to Γ → X. Note, the dispersions in the [001], [010] and [100] directions are the same for the isotropic fcc solid. For the AB alloy, we use the conventional unit cell with four atom basis and a simple cubic lattice to specify the unit cell. This description results in a simple cubic Brillouin zone with a length of *π*/*a*_0_, where *a*_0_ is the lattice constant. As the AB alloy are oriented in the [001] and[010] directions to form interfaces with the isotropic solid (as shown in Fig. 1b and c), the dispersion relations are analyzed in these two directions of high symmetry. The bulk phonon density of states are calculated by taking the Fourier transform of the velocity auto-correlation function and using the standard procedure for power spectral density calculations^43^. For each atom under consideration, a velocity fluctuation time series is obtained at 10 fs intervals for a total of 500 ps under the NVE integration at 30 K after the equilibration process. The phonon density of states for material A, material B and the AB (L1_0_) alloy in the cross-plane and in-plane directions are also plotted in Fig. 3 along side their respective phonon dispersion relations.

## Results and Discussions

### Lennard-Jones based structures

The wave-vectors are normalized by 2 *π*/*a*_0_ (*k* ^*^ = *k*/(2*π*/*a*_0_)) for all phonon dispersion relations shown in Fig. 3. For the LJ-based monatomic crystals, although the Brillouin zone extends to *k* ^*^ value of unity, the branches have been folded at *k* ^*^ = 0.5 to be consistent with the dispersions for the L1_0_ AB alloy. For comparison, we have also plotted the dispersion relation for the material A with the four atom conventional unit cell (similar to the calculations for the AB alloy) in the [001] direction. The additional dispersion curves (denoted by the blue diamond symbols in Fig. 3a) correspond to dispersion curves along other directions in the octahedron Brillouin zone (defined by the primitive unit cell) that arise due to the change in the shape of the Brillouin zone and are therefore mapped in the[001] direction of the conventional unit cell^44^. The phonon dispersion and DOS calculations for materials A and B display characteristics typical to fcc solids, whereas, the dispersions of the L1_0_ AB alloy in the in-plane and cross-plane directions are markedly different from the monatomic (isotropic) structures. The DOS for the AB alloy is also distinct from that of materials A and B with the formation of stop bands arising between frequencies of ~0.5 and ~1.3 THz, which is attributed to the two atom basis in the unit cell of the AB alloy (see Fig. 1). The acoustic phonons in the AB alloy extend up to 0.8 THz, and therefore the vibrational spectrum of material B with a cutoff frequency of *f* = 1 THz demonstrates a better spectral overlap with the acoustic phonons of the AB alloy. The vibrational spectra of Material A (which extends till ~2 THz) demonstrates an overall better spectral overlap with the AB alloy in terms of their cutoff frequencies since the optical phonons in the AB alloy extend from ~1 to 2 THz. Also, the group velocities, ${v}_{{\rm{g}}}=|\partial \omega /\partial k|$ of the optical phonons in the in-plane directions are higher than that in the cross-plane direction, the consequences of which will be discussed in terms of the thermal boundary conductance in the following paragraphs.

**Figure 4:**
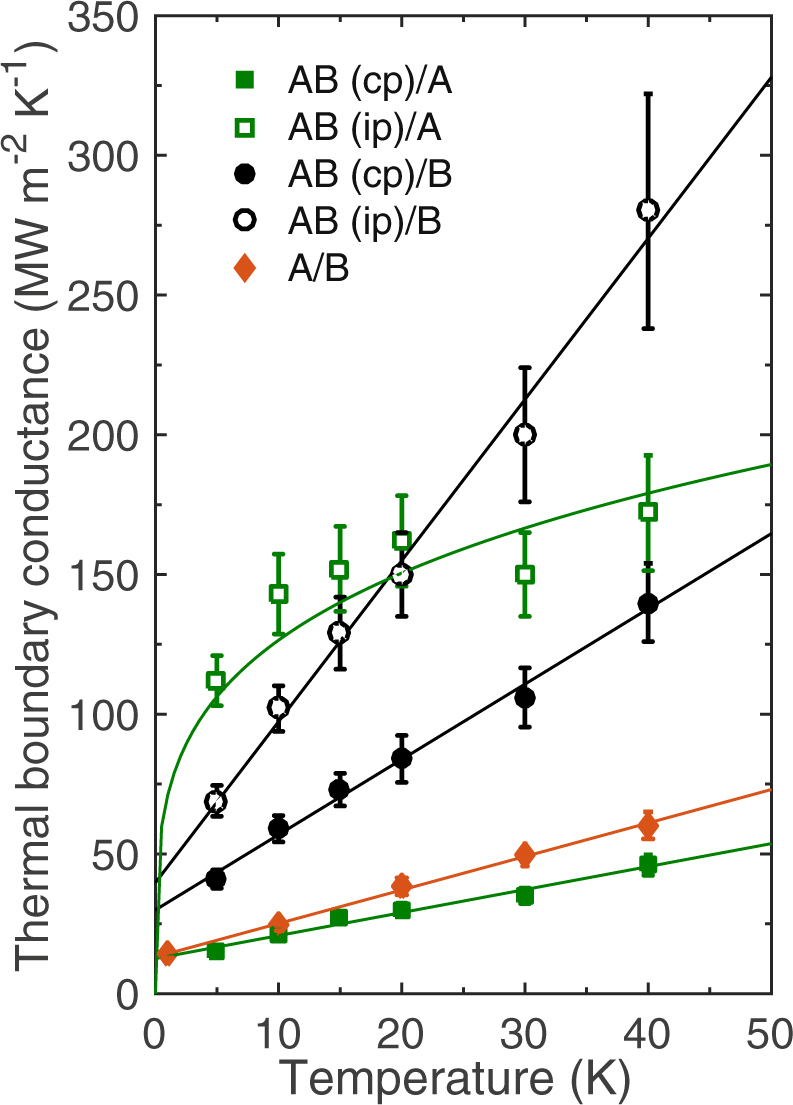
Temperature dependence of thermal boundary conductance across the various interfaces composed of the AB alloy and materials A and B.

Figure 4 shows the thermal boundary conductances as a function of temperature for interfaces comprised of the AB alloy in the in-plane and cross-plane directions with materials A and B. For comparison, *h*_K_ across the material A/material B interface is also plotted. The uncertainties in the MD-predicted *h*_K_ are calculated from three independent simulations and taking into consideration the uncorrelated fluctuations in the data sampling. For the A/B interface, we have considered the effect of different crystallographic orientations on *h*_K_ and found no correlation between the different crystal orientations within uncertainties of the MD-predictions. This result is in agreement with previous simulations and experimental results on cubic crystals where *h*_K_ does not depend on the relative orientation^23, 24, 45^.

For the interfaces comprised of the L1_0_ AB alloys, *h*_K_ demonstrates clear anisotropy with the in-plane direction showing considerably higher conductances as compared to the cross-plane direction for both AB/A and AB/B interfaces. In the cross-plane direction, the conductance across the AB/B interface is higher than the AB/A interface, even though material A has a better overlap in the DOS with the AB alloy as noted earlier. However, as material B has a better overlap with the acoustic phonons in the AB alloy, *h*_K_ across the interface comprising of AB alloy oriented in the cross-plane direction and material B is greater in comparison to that across the AB(cp)/A interface. This points to the fact that a better relative match between the cutoff frequencies (or Debye temperatures) of two materials does not necessarily result in a high conductance when the two materials have different crystallographic configurations and corresponding phonon dispersions. While the cross-plane conductances are higher for the case with the better match in the acoustic modes (across the AB(cp)/B interface), the conductances for the AB(ip)/A interface with better overall spectral overlap are relatively higher at lower temperatures. At higher temperatures (>20 K), the linear increase in *h*_K_ for the AB(ip)/B interface leads to higher conductances as compared to that for the AB(ip)/A interface (where the conductance at higher temperatures studied in this work are similar within uncertainties).

The temperature dependence for the AB(ip)/A interface is unique as all other interfaces considered show a linear increase in *h*_K_ (c.f. Fig. 4). Along these lines, the A/B interface demonstrates a linear increase in *h*_K_, which is in agreement with the results from prior studies that have considered LJ argon/“heavy-argon” interfaces (refs 46 and 47). These prior studies have attributed the linear increase in *h*_K_ with temperature to anharmonic effects in the bulk and at the interface, which enhances transmission of phonon frequencies that exceed the cutoff frequency of the heavier solid. Until recently, it was largely assumed that the bulk properties of the two materials adjoining the interface (such as the spectral overlap between the two materials) could sufficiently describe phonon transport across interfaces. However, Gordiz and Henry have shown that the vibrational properties of the interface (which are dictated by the two materials that form the interface) need to be considered in order to properly describe the energy transport mechanisms occurring at the interface^48, 49^.

Generally, the linear increase in *h*_K_ with temperature is related to multiple-phonon inelastic scattering events at the interface^3, 50, 51^. This would suggest that the ability of inelastic channels to increase interfacial conductance for our AB(ip)/A interface saturates at ~20 K for the temperature range studied in this work. Although, the optical modes in the AB alloy are “elastically accessible” to material A due to the DOS overlap in both cases when the AB alloy is oriented in-plane or in the cross-plane directions, one of the reasons for the difference in the large anisotropy in *h*_K_ might potentially be due to higher group velocities of the optical modes in the in-plane direction as compared to that in the cross-plane direction.

**Figure 5:**
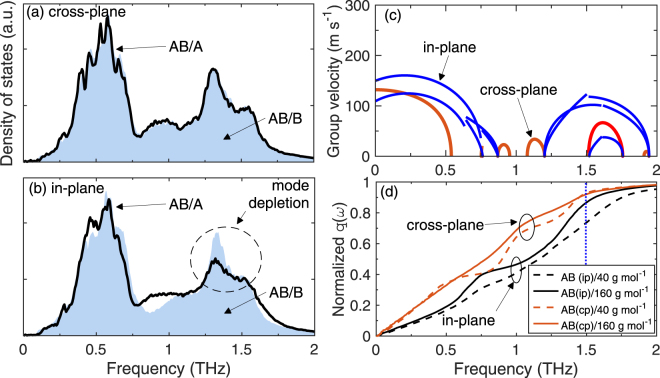
Local phonon density of states of atoms in the bilayer in the AB alloy immediately adjacent to the interface comprised of the AB alloy in the (**a**) cross-plane and (**b**) in-plane direction and materials A and B. Considerable mode depletion is observed for frequencies in the range of 1.25 to 1.75 THz for the AB(ip)/B interface as compared to all other interfaces. (**c**) Group velocity as a function of frequency calculated for the modes in the in-plane and cross-plane directions for the AB alloy. (**d**) Normalized heat current accumulation from the AB alloy to the cubic solid across the interface for the two different orientations of the AB alloy.

To understand the differing temperature trends in more detail, we calculate the DOS of atoms in the bilayer of the AB alloy that are immediately adjacent to the interface for the two orientations (c.f., Fig. 5a and b). Even though the DOS are calculated for bilayers directly adjacent to different materials in two different orientations, a drastic deviation from the bulk DOS is not observed for all cases. However, for the AB(ip)/A case, a mode depletion is observed at the frequency interval of 1.25 to 1.75 THz. This frequency range corresponds to the optical phonon modes that demonstrate higher group velocities in the in-plane direction as compared to that in the cross-plane direction as noted earlier. This is quantitatively depicted in Fig. 5c, which shows the calculations of group velocities as a function of frequency in the in-plane and cross-plane directions. The mode depletion that is observed in only one direction (Fig. 5b) suggests that the acoustic phonons in material A are coupled to the optical phonons in the AB alloy oriented in the in-plane direction more so than the cross-plane direction. This is because the phonon energy of these modes are distributed between the two solids, which leads to the diminished DOS of these modes in the bilayer of the AB alloy adjacent to the interface.

To quantitatively support the observations presented in the above paragraph, we turn to the spectral decomposition method at the interface (the details of which are presented in our previous work)^20^ to investigate which modes carry the significant amount of heat across the interface. Briefly, the heat flux is spectrally resolved by the relation^46^, $Q={\int }_{0}^{\infty }\frac{d\omega }{2\pi }q(\omega )$, where *ω* is the angular frequency and *q*(*ω*) is the spectral heat current. For pairwise interactions between an atom *i* and *j*, the heat current is proportional to the correlation between the interatomic force ${\vec{F}}_{ij}$ between the atoms and the velocities, ${q}_{i\to j}(\omega )\propto \langle {\vec{F}}_{ij}\cdot ({\vec{v}}_{i}+{\vec{v}}_{j})\rangle$, where the brackets denote steady-state nonequilibrium ensemble average^52–54^. To this effect, under the NVE integration at 30 K, velocities of atoms near the interface for the AB alloy are tabulated for a total of 10 ns with 10 fs time intervals along with the forces due to atoms on the other side of the interface. The resulting heat current accumulations from the AB alloy to the other side of the interfaces are shown in Fig. 5d. As is clear, the high frequency optical modes in the in-plane orientation of the AB alloy contribute more towards thermal boundary conductance than in the cross-plane orientation. Moreover, for the AB(ip)/A interface, the modes >1.5 THz contribute ~30% of the heat flux, whereas for the other three cases, the relative contribution to the heat current from these modes is considerably lower (<10%). This is in line with the observation of mode depletion observed for the AB(ip)/A interface (while for the other three cases, the interfacial DOS is similar to that of the bulk) as discussed in the above paragraph and shown in Fig. 5a and b.

**Figure 6:**
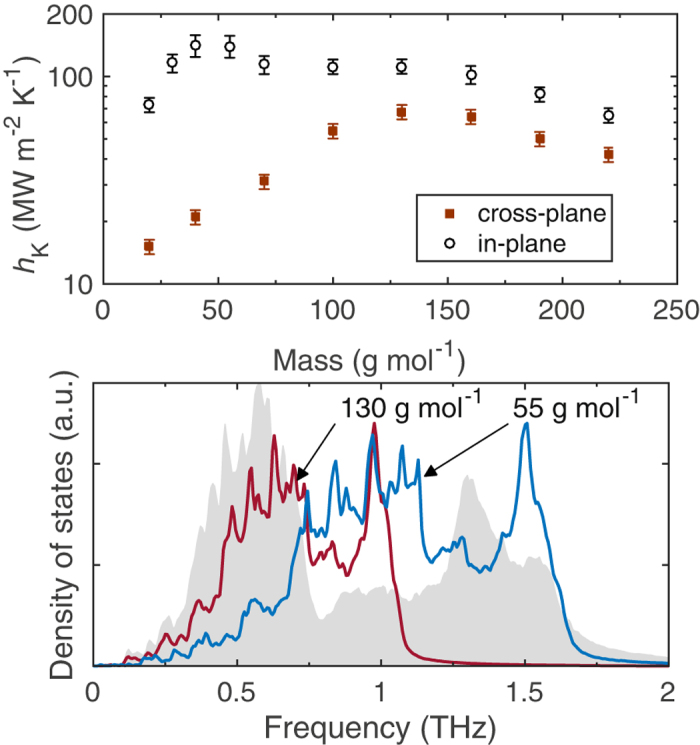
(**a**) Thermal boundary conductance for interfaces comprised of various masses of the LJ sytems of homogeneous fcc solids and the AB alloys arranged in the cross-plane (solid squares) and in-plane (open circles) directions at the interface. (**b**) Characteristic bulk density of states for the AB alloy, and the homogeneous fcc solids with *m* = 130 and 55 g mol^−1^.

To better understand the role of relative match of the cutoff frequencies (or Debye temperatures) on the interfacial heat flow, we performed additional simulations by varying the mass of the isotropic fcc solid while the AB alloy remains unchanged; note, increasing the mass decreases the cutoff frequency via the relation, $\omega \propto \mathrm{1/}\sqrt{m}$. Figure 6a shows *h*_K_ as a function of mass of the fcc solid for interfaces comprised of in-plane and cross-plane configurations of the AB alloy. For the cross-plane configuration, *h*_K_ increases monotonically up to a mass of 130 g mol^−1^ and decreases thereafter. In Fig. 6b the DOS for the solid with *m* = 130 g mol^−1^ is plotted along with the DOS for the AB alloy. For comparison, we also plot the DOS for the solid with *m* = 55 g mol^−1^, which represents a lighter solid with a broader spectrum of frequencies that can elastically accommodate the optical phonons in the AB alloy. The acoustic modes in the heavier solid (with *m* = 130 g mol^−1^) better overlap the acoustic modes in the AB alloy, which results in an increase in *h*_K_ when the AB alloy is oriented in the cross-plane direction. This suggests that for interfaces comprised of AB alloys in the cross-plane configuration, interfacial heat flow is mainly mediated through acoustic phonons. However, a further increase in the mass leads to a mismatch in the heat carrying acoustic phonons between the solids, which could potentially be one of the reasons for the decrease in *h*_K_. In contrast, for the in-plane configuration, the maximum in *h*_K_ is observed for *m* = 55 g mol^−1^, which has a better overall spectral overlap. In this case, the high frequency acoustic modes in the fcc solid coincide with the high frequency optical modes in the AB alloy (in the 1.25 to 1.75 THz range). Going back to the phonon dispersion relations and group velocities shown in Figs 3b,c and 5c, the optical modes in the in-plane configuration have a higher group velocity as compared to that in the cross-plane direction and therefore carry a more significant proportion of heat. This manifests in a higher *h*_K_ when the optical modes are “elastically accessible” to the acoustic modes in the fcc solid across the interface, as noted earlier. It is also worth noting that the anisotropy in *h*_K_ is pronounced for comparatively lighter masses with broader frequency spectrums that can elastically couple with the optical phonons in the AB alloy.

### Stillinger-Weber based structures

**Figure 7:**
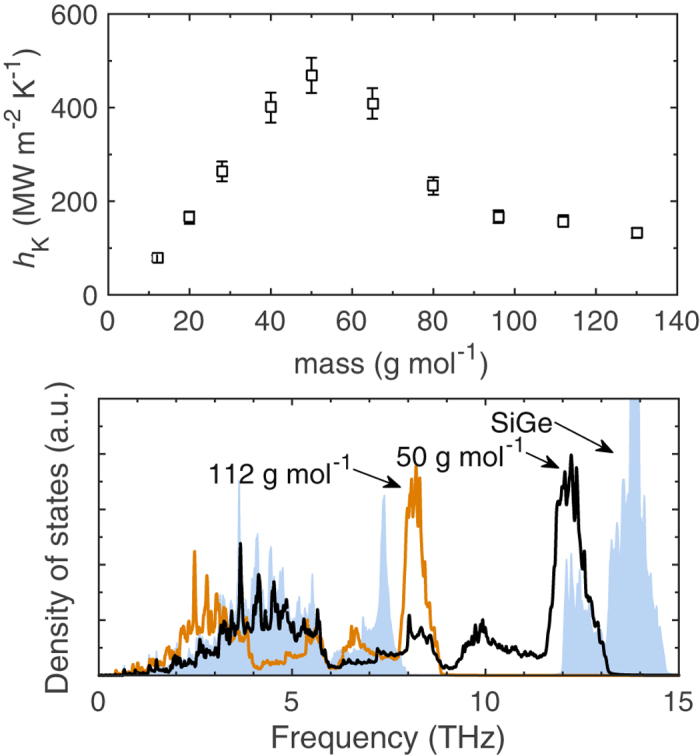
(**a**) Thermal boundary conductance for interfaces comprised of various masses of the homogeneous solid arranged in a diamond cubic crystal structure and the zincblende structure with the two-atom basis (solid squares). (**b**) Characteristics bulk density of states for the SiGe alloy, and the homogeneous solids with *m* = 112 and 50 g mol^−1^.

As most of the semiconductor devices are based on Si, we also investigate the role of optical phonons across interfaces comprised of diamond cubic crystal structures defined by the SW potential. The AB alloy in this case is formed from a combination of Si and mass-heavy Si with *m* = 72.6 g mol^−1^ (which we refer to as Ge) arranged in a 1 × 1 layered structure with the atoms placed in a fcc lattice with a 2 point basis with a_0_ = 5.44 Å, which we have referred to as the zincblende structure. Similar to the LJ structures, we vary the mass of the homogeneous crystal (forming the interface with the SiGe alloy) to gauge the effect of DOS overlap (of the optical phonons) between the two materials. Figure 7a shows the results of the NEMD calculations and Fig. 7b shows representative DOS for the SiGe alloy and the homogeneous diamond structures with *m* = 50 and 112 g mol^−1^. Similar to the dependence of *h*_K_ on the mass of material B for the AB(cp)/B interfaces in the LJ systems, *h*_K_ increases linearly up to 50 g mol^−1^ and decreases thereafter. As is clear from Fig. 7b, the acoustic phonons in the 50 g mol^−1^ perfectly coincide with the acoustic phonons in the SiGe alloy. This suggests that acoustic phonons are the primary heat carriers mediating interfacial transport across these diamond structured materials. This is in line with the modal decomposition of thermal boundary conductance across Si/Ge interfaces carried out in ref. 55 where it is shown that ~80% of the heat is carried by frequencies that fall in the acoustic spectrum of Si.

**Figure 8:**
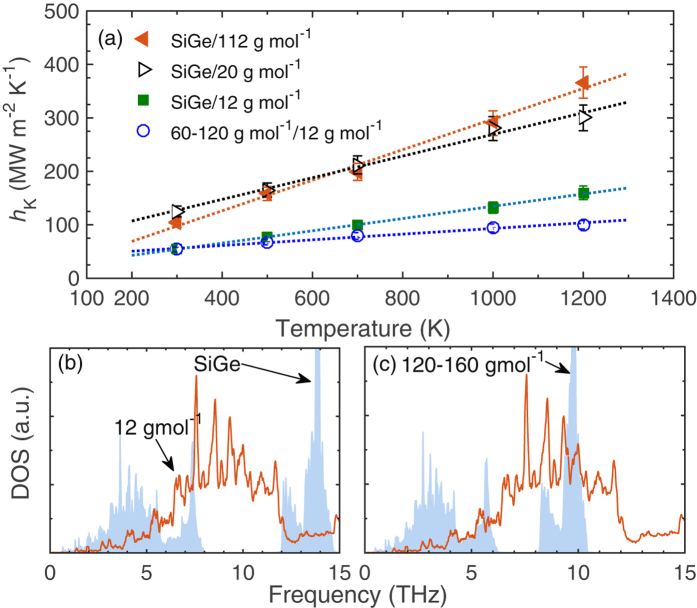
(**a**) Thermal boundary conductance as a function of temperature for the various interfaces comprised of the diamond cubic crystals. Bulk phonon density of states for the (**a**) SiGe and (**b**) AB alloy with *m*_A_ = 120 and *m*_B_ = 160 g mol^−1^. For comparison the frequency range of the acoustic modes for the solid with *m*_A_ = 12 g mol^−1^ is also shown.

Figure 8a shows the temperature dependence of *h*_K_ for interfaces comprised of solids with *m* = 112 and 20 g mol^−1^ and the SiGe alloy. Within uncertainties, *h*_K_ values for the two interfaces are similar for almost the entire temperature range. However, at 1400 K, *h*_K_ is greater for the interface comprised of *m* = 112 g mol^−1^ due to the relatively stronger anharmonic interactions of the acoustic phonons that are better overlapped with the acoustic phonons of the SiGe alloy as compared to that of the solid with *m* = 20 g mol^−1^.

To investigate the effect of acoustic phonons in the homogeneous solid coupling with the high frequency optical phonons in the AB alloy, we run additional simulations on interfaces comprised of SiGe alloy and solid with *m* = 12 g mol^−1^ and an AB alloy with the two atoms defined by *m* = 120 g mol^−1^ and *m* = 160 g mol^−1^. The band gap for the alloy with the lower mass ratio between the atoms is relatively smaller as compared to that of the SiGe (c.f., Fig. 8b and c for the DOS comparison between the alloys), allowing for the overlap of the acoustic phonons in the solid with *m* = 12 g mol^−1^ with the high frequency phonons in the AB alloy. The results for these interfaces are also plotted in Fig. 8a, which show that *h*_K_ at low temperatures (<700 K) are similar for the two cases, whereas, the increase in anharmonic interactions at the interface comprised of the SiGe alloy leads to greater conductances as compared to the case where the acoustic phonons in the homogeneous solid are coincidental with the high frequency optical phonons in the AB alloy at higher temperatures. It is also worth noting that the interfaces composed of diamond and zincblende structures demonstrate linear temperature dependencies for the temperature range studied, even when the acoustic phonons are “elastically accessible” to the high frequency optical phonons, which is unlike in the interfaces formed between the fcc and L1_0_ structures where the conductance of the AB(ip)/A interface deviates from the linear dependence (c.f., Fig. 4). Similar linear temperature trends of *h*_K_ for interfaces comprised of an fcc solid and GaN (with wurtzite crystal structure) were observed in ref. 22. For these structures, the better overlap of acoustic phonons of the fcc solid to the acoustic modes in the GaN was shown to enhance the conductance^22^.

**Figure 9:**
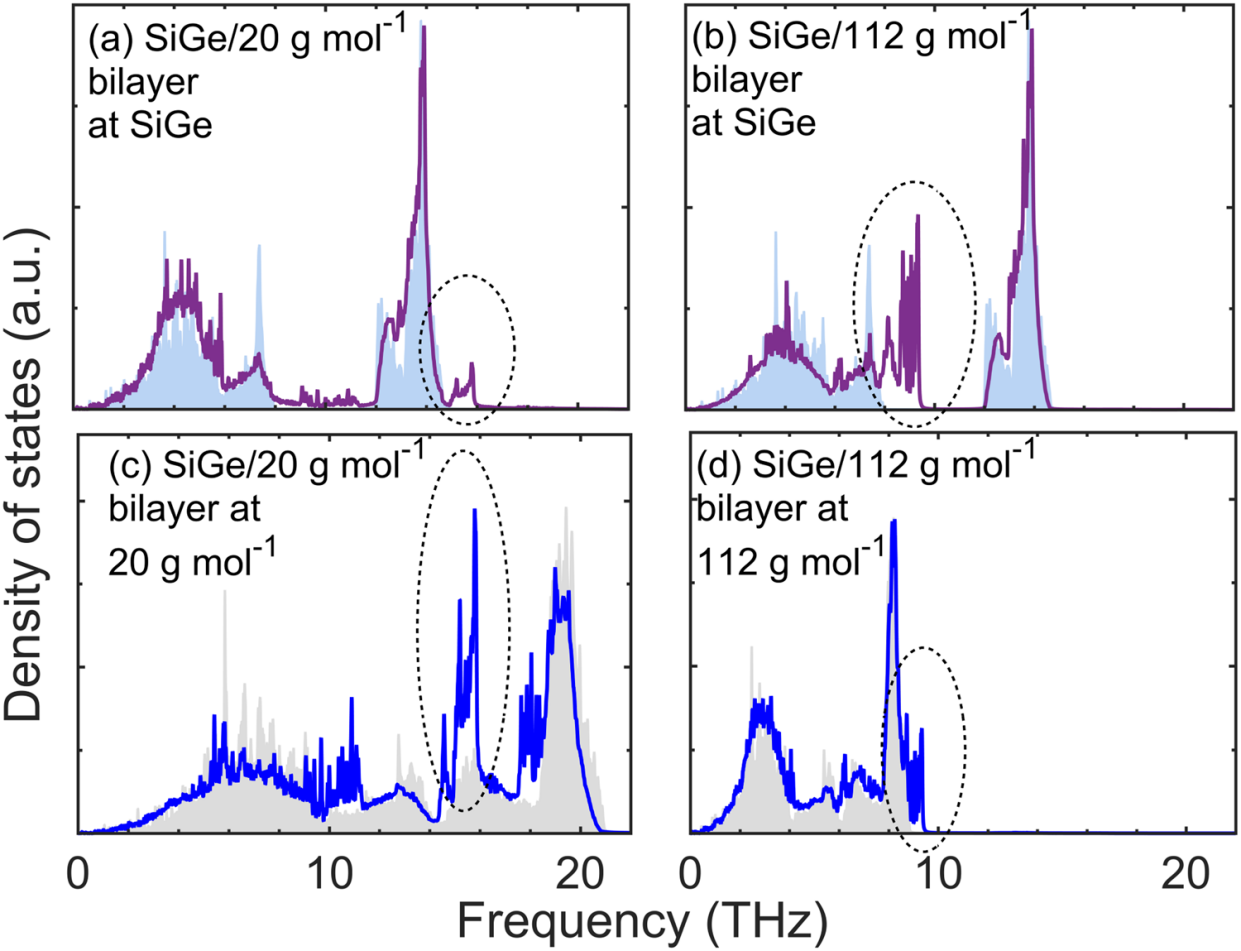
Local phonon density of states of atoms within bilayers immediately adjacent to the interfaces comprised of the SiGe alloy and diamond cubic structures defined by *m* = 112 and 20 g mol^−1^. The presence of interfacial modes with frequencies greater than the cutoff frequency of the heavier solid near the vicinity of the interfaces are highlighted in the figures.

We now study the local phonon DOS immediately adjacent to the interfaces for our diamond cubic structures to better understand the results discussed in the previous paragraphs. To this end, we calculate the DOS of bilayers for the SiGe alloy and diamond structures with *m* = 20 g mol^−1^ and *m* = 112 g mol^−1^ that comprise the interface, the results of which are plotted in Fig. 9. Unlike the LJ systems, the local DOS reveals the existence of interfacial modes in two materials that are absent in the bulk DOS for each of the corresponding material. These interfacial modes are highlighted in Fig. 9. It is interesting to note that these modes are above the cutoff frequency for either the SiGe alloy (for the interface comprised of SiGe and diamond structure with *m* = 20 g mol^−1^) or the homogeneous diamond structure (for the interface comprised of SiGe and diamond structure with *m* = 112 g mol^−1^). This suggests that the contributions to *h*_K_ from these modes are a direct result of inelastic energy exchange due to the anharmonicity at the interface. Similar results for interfaces comprised of Si and Ge have also been reported, where considerable amount of heat is carried by interfacial modes at 12–13 THz region that are beyond the cutoff frequency of Ge^55–57^. The large DOS for the interfacial modes is also not an artifact of the SW potential used in this work as similar results (for DOS calculations as shown Fig. 9) are obtained when the atomic interactions are defined by the Tersoff potential^58, 59^. These results suggest that the description of heat transfer across these interfaces cannot be accurately depicted by only considering the bulk phonon properties of the two materials and it becomes necessary to consider the localized and nondispersive interfacial modes to accurately describe interfacial heat transfer across these diamond cubic structures. Moreover, Gordiz and Henry postulate that these interfacial modes facilitate interfacial energy transport among other phonon modes since the conductance decreases considerably when these interfacial modes are excluded (see Fig. 5 of ref. 55). For a detailed investigation of the role of these interfacial modes on heat transfer across Si/Ge interfaces, the reader is referred to ref. 55. We note that the fcc structures defined by the LJ potential did not show similar existence of pronounced interfacial modes that mediate thermal transport. Moreover, in ref. 60, an interface comprised of InGaAs/InP (note, InGaAs and InP both exhibit zincblende crystal structures) was also shown to exhibit little to no interfacial modes that mediate thermal transport across these materials. This suggests that the existence of large interfacial modes could be unique to interfaces composed of diamond cubic crystal structures of Si and Ge.

## Conclusion

We assessed the role of optical phonons on the thermal boundary conductance across materials with different crystal structures and orientations. We show that for interfaces formed between a fcc solid and a L1_0_ solid (where the L1_0_ solid exhibits alternating atomic layers in certain orientations), optical phonons are preferentially better coupled for the case when the interface is comprised of the layered crystal oriented in the direction which possesses higher group velocities of these modes, thus increasing the overall thermal boundary conductance. In contrast, interfaces comprised of diamond cubic crystal structures demonstrate higher conductances for interfaces formed with a better overlap of acoustic phonons on either side. At high temperatures where anharmonic interactions become important, acoustic phonons directly coupling with high frequency optical phonons is shown to lower the overall conductance for these structures, which is in contrast to the results obtained for the LJ-based solids. Furthermore, the presence of localized interfacial optical modes, which in this work are unique to the diamond cubic crystal structures are shown to mediate thermal conductance across these interfaces.
